# Bioethics, globalization and pandemics

**DOI:** 10.1080/11287462.2021.2011006

**Published:** 2022-02-06

**Authors:** Gustavo Ortiz-Millán

**Affiliations:** Instituto de Investigaciones Filosóficas, Universidad Nacional Autónoma de México, Mexico City, Mexico

**Keywords:** Global bioethics, globalization, COVID-19, zoonosis, inequality

## Abstract

Bioethics should pay more attention to globalization and some of its consequences than it has done so far. The COVID-19 pandemic would not have been possible without globalization, which has also increased some of its negative consequences. Globalization has intensified wildlife trade in the world. One of the main hypotheses about the origin of this pandemic is that it originated in illegal forms of wildlife trade in China. In the last 30 or 40 years, there have been zoonotic outbreaks at a much frequent pace than before, many of those have been related to wildlife trade. Legal and illegal wildlife trade has grown in the shadow of globalization. Second, globalization has had a huge impact on the redistribution of wealth in the world. Since 1990 income inequality has increased in most high- and in many middle- and low-income countries. A country’s level of pre-COVID income inequality is the best predictor of the COVID death rate. These two issues are not unrelated. People living in poverty in LMIC tend to suffer more from infectious diseases and tend to be marginalized from the health sector. Additionally, poverty tends to reproduce the conditions under which zoonotic diseases can more easily spread.

The first issue of *Global Bioethics* was published in 1988, when the words “global” and “globalization” had completely different connotations to the ones they have today. Just consider that only one year later, in 1989, a wave of political revolutions took place in Eastern Europe, the Berlin Wall fell and the whole process culminated in 1991 with the dissolution of the Soviet Union, one of the two world superpowers at that time. This also brought the Cold War to an end. These events led Francis Fukuyama to announce the “end of history,” by which he meant that since communism and other forms of authoritarianism had been defeated, there was no serious competition for liberal democracy and market economy. To be sure, the “end of history” is a controversial idea, but Fukuyama may have been right in proclaiming market economy’s triumph, which – along with the digital revolution – meant the starting point of a new form of globalization. Globalization is not a new phenomenon in history, but in each period it has had different economic, political, cultural, environmental and health dimensions. The form of globalization that begins in the 1980s has its own characteristics in all these dimensions, and that’s why it poses new and different bioethical problems.

This new form of globalization has been defined as “the expansion and intensification of social relations and consciousness across world-time and world-space” (Steger, 2013, p. 35). This means much more interconnected and interdependent relations between individuals, non-governmental organizations, corporations and countries around the world. Now, what happens in one country may have been shaped by events occurring at the same time somewhere at the other side of the world. But these interdependency and interconnections come at a price.

Macklin (2021) is right in pointing to the COVID-19 pandemic as an illustration of this global interrelatedness. She highlights how some relations between and among countries have taken a new light under the pandemic, and how new situations that call for global collaboration and new problems have emerged. I want to call attention to some other aspects of the current pandemic in which a more globalized world presents bioethics with new challenges; cases that call for a global response and also a global bioethics.

## Zoonotic outbreaks and globalization

In a way, this pandemic would not have been possible without globalization. One of the main hypotheses about the origin of this pandemic is that it had a zoonotic origin, and it is very likely that it originated in illegal forms of wildlife trade in China (Standaert, 2020). In wildlife trade animals are usually kept under conditions that provoke immunosuppression, predisposing them to infections and other diseases that may eventually be transmitted to humans. Legal and illegal wildlife trade has grown in the shadow of globalization: since 1980 worldwide legal wildlife trade has increased by 2000% (Kukreti, 2020). It is hard to know how much illegal trade has grown in the same period since it is a clandestine phenomenon, but we do know, for instance, that rhino poaching has increased 7700% from 2007 to 2013 (WWF, 2021). Nowadays, almost every country in the world takes part in this very lucrative business, either as exporters or importers (UNODC, 2020). Zoonotic outbreaks due to wildlife trade have been potentiated by the relative ease of buying wildlife from almost anywhere in the world (CRS, 2021).

If previous outbreaks of epidemic diseases and infections were limited to more or less specific geographical regions, today globalization has made it easier for these diseases to spread more rapidly throughout the world. Today, more than ever before, more people travel to and from risk regions, thereby contributing to the spread of pathogens. In a matter of days, any emerging disease can travel from previously inaccessible regions to major cities at the other side of the world.

Other recent zoonotic outbreaks have had an environmental origin, related to the invasion of habitats and to the close contact that the natural reservoir fauna is forced to have with domestic animals and, ultimately, with humans. They also have to do with biodiversity loss and the increasing prevalence of certain pathogens in reservoir populations (Keesing & Ostfeld, 2021). This biodiversity loss has also been intensified by globalization, since there is an increasing global demand for agricultural products – particularly animal products – which require the deforestation of huge portions of tropical forests. This, in its turn, intensifies climate change, one of the other major causes of biodiversity loss.

According to the United States Centers for Disease Control and Prevention (CDC), three out of every four new or emerging infectious diseases in people come from animals (CDC, 2017). The rate at which zoonotic outbreaks have appeared in the last 40 years or so has been greater than ever before (Quammen, 2014). Epidemiologists and other experts have warned us that COVID-19 is not the last zoonotic disease we are going to see, that there may be some other epidemics in the near future (UNEP, 2020), so we should be better prepared to prevent them.

Climate change, wildlife trade, biodiversity loss and zoonotic outbreaks are global problems that require global solutions. Of course, we need more effective zoonotic disease surveillance systems if we want to prevent future pandemics, but we also need to reconsider our relations with nature and especially with wildlife. Global bioethics should take the concept of One Health more seriously; this is the approach that recognizes that human health is deeply interconnected with the health of animals, and ecosystems (WHO, 2017). Global bioethics should take a crucial role in this task.

## Globalization, inequality and pandemics

Globalization has had a huge impact on the redistribution of wealth in the world. Some of this redistribution has been positive and many countries have gained in the process. For instance, some 25 years ago, the average standard of living in high-income countries such as France or Germany was twenty times higher than China, India or other Asian economies. Today this gap has been cut in half and people have much better living conditions (Bourguignon, 2015, p. 2). However, even if inequality among countries has improved in this period, globalization has not necessarily benefited everyone within countries and in many cases has brought about more inequality. Income inequality has become worse: since 1990 income inequality has increased in most high-income countries and in many middle- and low-income countries (LMIC). Today, 71% of the world’s population lives in countries where inequality has grown during this period (UN, 2020). Even in high-income countries, such as the US, income inequality has increased by about 20% from 1980 to 2016 (Horowitz et al., 2020). Around 1980, the top 1% of US income earners received only 12% of the nation’s income; by 2007 the top 1% get in one week 40% more than the bottom fifth receive in a year; the top 0.1 percent received in a day and a half about what the bottom 90% received in a year; and the richest 20% of income earners earn in total after tax more than the bottom 80% combined (Stiglitz, 2012). In different degrees, but this situation has been going on in many countries in the world (Bourguignon, 2015).

Income inequality has been associated with disparities in life expectancy. In the US in the 1970s, the life expectancy of a high-income 60-year-old man was 1.2 years longer than that of a man in the bottom half of the income distribution; by 2001, the difference was of 5.8 years longer (Waldron, 2007). Between 2001 and 2014, life expectancy increased by 2.34 years for men and 2.91 years for women in the top 5% of the income distribution, but increased by only 0.32 years for men and 0.04 years for women in the bottom 5%. In 2014, the gap in life expectancy between the richest 1% and poorest 1% of individuals was 14.6 years for men and 10.1 years for women (Chetty et al., 2016). It is likely that this gap will grow as a result of the pandemic.

When trying to explain why some regions are losing many more lives than others during the pandemic, Martin Eichenbaum and his colleagues discarded variables such as age, the share of doctors per 1000 people, the size of the average household, the proportion of urban to rural areas, and average temperature, and discovered that a country’s level of pre-COVID income inequality is the best predictor of the COVID death rate (Eichenbaum et al., 2021). Variations in COVID-19 death rates tend to occur in places with higher pre-COVID income inequality. In these places, the poor have a disproportionately higher mortality rate due to COVID-19 basically because of disparities in access to quality healthcare and in health preconditions that predate the pandemic. The pandemic has accentuated inequalities in life and death. In fact, the pandemic has affected the low-income population not only because of their lack of access to quality health services, but also because they have lower levels of health on average because they tend not to have health coverage, work in low wage jobs, many live in polluted areas and, in that sense, they may be more susceptible to being affected by epidemic diseases.

Many low-income people do not have health insurance. According to recent research about the impact of lack of healthcare coverage in the US, “each 10% increase in the proportion of a county’s residents who lacked health insurance was associated with a 70% increase in COVID-19 cases and a 48% increase in COVID-19 deaths” (Dorn & Gordon, 2021). Without health coverage, low-income people tend to delay seeking medical care or forgo care because of economic reasons; many people live on a day-to-day basis so cannot afford not to work; this allows disease to spread undetected to family members, neighbors, co-workers, etc. So inequalities in income and in healthcare coverage tend to accelerate the spread of the pandemic. When there are huge income- and health-inequalities in a society, then not only low-income people are at risk, the whole society is at risk.

## Conclusions: what a global bioethics should do

I have tried to illustrate the global nature of some problems generated by the pandemic resorting to two cases: zoonoses and income inequality. These may seem to be unrelated problems, but they are not. People living in poverty in LMIC tend to suffer more from infectious diseases, mostly zoonoses such as some malarian infections, dengue, chagas, chikungunya and different kinds of influenza, among others. These people tend to be marginalized from the health sector, and big pharmaceutical companies usually neglect diseases that affect the poorest, because they are less commercially attractive (Smedley, 2015), condemning them to a cycle of poverty. Additionally, poverty tends to reproduce the conditions under which many of these diseases can more easily spread (Seimenis, 2012). Poverty is also one of the most important ingredients contributing to wildlife hunting and trade (Duffy et al., 2016). In a globalized world, we can discover that many problems that do not seem to be related are in fact connected and are part of a complex network. A global bioethics should help us in the task of connecting the dots.

A global bioethics should also help us understanding how global processes affect actions and policies of individual nations. Failure to take this global perspective would only give us a partial view of these phenomena. Global bioethics should be more integrated in the growing field of global studies, the interdisciplinary study of global macro-processes, focusing more on issues relating to economic and cultural globalization, and its effects on the global environment, rather than on nation states as the unit of analysis. As Macklin tells us, this new definition of global bioethics should also encompass “the study of cooperation and collaboration (and lack thereof) between and among nations. It embraces questions about the role of international organizations such as [the World Health Organization] and the influence they may exert over actions or policies of individual nations” (Macklin, 2021). By taking this approach, bioethics is going to be in a much better position to suggest global solutions to our global problems. To begin with, it should help us in ending the current pandemic and in preventing future ones.
